# Antioxidant, antimicrobial and healing properties of an extract from coffee pulp for the development of a phytocosmetic

**DOI:** 10.1038/s41598-024-54797-0

**Published:** 2024-02-23

**Authors:** Érica Mendes dos Santos, Lucas Malvezzi de Macedo, Janaína Artem Ataide, Jeany Delafiori, João Paulo de Oliveira Guarnieri, Paulo César Pires Rosa, Ana Lucia Tasca Gois Ruiz, Marcelo Lancellotti, Angela Faustino Jozala, Rodrigo Ramos Catharino, Gisele Anne Camargo, Ana Cláudia Paiva-Santos, Priscila Gava Mazzola

**Affiliations:** 1 Faculdade de Ciências Farmacêuticas, Universidade de Campinas (UNICAMP), Rua Cândido Portinari, 200, Campinas, São Paulo 13083-871 Brazil; 2 Faculdade de Ciências Médicas, Universidade de Campinas (UNICAMP), Rua Tessália Vieira de Camargo, 126, Campinas, São Paulo 13083-887 Brazil; 3 Laboratory of Industrial Microbiology and Fermentation Process (LAMINFE), University of Sorocaba, Sorocaba, São Paulo 18023-000 Brazil; 4 Institute of Food Technology, ITAL, Av. Brasil, 2880, Campinas, São Paulo 13070-178 Brazil; 5 Department of Pharmaceutical Technology, Faculty of Pharmacy of the University of Coimbra, University of Coimbra, Pólo das Ciências da Saúde, Azinhaga de Santa Comba, 3000-548 Coimbra, Portugal; 6 REQUIMTE/LAQV, Department of Pharmaceutical Technology, Faculty of Pharmacy of the University of Coimbra, University of Coimbra, Azinhaga de Santa Comba, 3000-548 Coimbra, Portugal

**Keywords:** Antioxidant, Antimicrobial, By-products, Coffee, Phytocosmetic, Wound healing, Drug discovery, Plant sciences

## Abstract

Consumer demand for natural, chemical-free products has grown. Food industry residues, like coffee pulp, rich in caffeine, chlorogenic acid and phenolic compounds, offer potential for pharmaceutical and cosmetic applications due to their antioxidant, anti-inflammatory, and antibacterial properties. Therefore, the objective of this work was to develop a phytocosmetic only with natural products containing coffee pulp extract as active pharmaceutical ingredient with antioxidant, antimicrobial and healing activity. Eight samples from *Coffea arabica* and *Coffea canephora* Pierre were analyzed for caffeine, chlorogenic acid, phenolic compounds, tannins, flavonoids, cytotoxicity, antibacterial activity, and healing potential. The Robusta IAC—extract had the greatest prominence with 192.92 μg/mL of chlorogenic acid, 58.98 ± 2.88 mg GAE/g sample in the FRAP test, 79.53 ± 5.61 mg GAE/g sample in the test of total phenolics, was not cytotoxic, and MIC 3 mg/mL against *Staphylococcus aureus*. This extract was incorporated into a stable formulation and preferred by 88% of volunteers. At last, a scratch assay exhibited the formulation promoted cell migration after 24 h, therefore, increased scratch retraction. In this way, it was possible to develop a phytocosmetic with the coffee pulp that showed desirable antioxidant, antimicrobial and healing properties.

## Introduction

Consumers are increasingly aware and are looking for products free of harmful chemicals. The trend in recent years has been to increase the use of products with natural ingredients from mineral, vegetable, or marine sources. Natural ingredients can be used in a formulation as active ingredients, preservatives, surfactants, among others^1^.

The food industry generates residues that could be used in pharmaceutical and cosmetic industry due to its properties. Among these residues, coffee pulp that is generated during cherry coffee processing into green coffee, stands out. In addition to the pulp, during wet and dry processing, by-products are generated, such as skin, mucilage, parchment and silverskin^2^. Pulp is the most by-product generated in volume during wet processing and for every 2 tons of coffee that are produced, 1 ton is pulp^3^. The main coffee species traded worldwide are *Coffea arábica* L. (arabica) and *Coffea canephora* Pierre (robusta)^4^.

Previous studies have shown that coffee pulp has active substances such as caffeine, chlorogenic acid, tannins, phenolic compounds, flavonoids, among others^5^. These components are responsible for its biological activities, such as antioxidant^6^, anti-inflammatory and antibacterial^7^. Based on these characteristics of coffee pulp, a topical product that would benefit wound healing process was thought.

Wound healing is a complex dynamic process that results in restoration of continuity and anatomical function of the skin, considered a regular biological process of the body, since skin has the natural ability to promote its regeneration^8^. This process occurs in four phases: hemostasis, inflammation, proliferation and remodeling^9^. Free radicals are generated during the wound healing process, and they have physiological effects on cell migration, angiogenesis and combating microorganisms that lodge in the wound. However, when the level of free radicals becomes high, damage to the cells and tissues surrounding the wound occurs^10^.

Therefore, the objective of this work was to develop a phytocosmetic produced only with natural products approved by Associação de Certificação Instituto Biodinâmico (IBD) containing coffee pulp extract as active pharmaceutical ingredient (API) with antioxidant, antimicrobial and healing activity. To this end, eight coffee pulp extracts were analyzed for caffeine, chlorogenic acid, phenolic compounds, tannins, flavonoids, cytotoxicity, antibacterial activity, and healing potential. The best extract was then incorporated into a phytocosmetic, which was characterized and analyzed for stability.

## Materials and methods

### Material

Caffeine (99% of purity), 1,1-diphenyl-2-picrilhhydrasyl and quercetin (95% of purity) were provided by Sigma-Aldrich (São Paulo, Brazil), methanol and aluminum chloride by Synth (São Paulo, Brazil), Folin–Ciocalteau, ethanol, gallic acid, algin, benzoic acid, glycerin and sodium hydroxide by Dinâmica (São Paulo, Brazil), acetonitrile by J.T. Baker (Phillipsburg, New Jersey, United States), acetic acid, sodium carbonate, tannic acid, lecithin, cetyl alcohol and sorbic acid by Êxodo Científica (Sumaré, São Paulo, Brazil), chlorogenic acid (95% of purity) by Fluorochem (Hadfield, United Kingdom) and persea gratissima (avocado) oil by Croda (Campinas, São Paulo, Brazil). All solvents and reagents used were of analytical grade.

### Coffee pulp

The coffee pulp was provided by the Institute of Food Technology (ITAL, Campinas SP, Brazil), through the NIT APTA (Agência Paulista de Tecnologia dos Agronegócios)^11^ and was registered in the National System of Genetic Resource Management and Associated Traditional Knowledge (SisGen) under register number AC8EF83. These samples were obtained from arabica and robusta species and were processed generating different types for analysis: 1. Arabica—extract; 2. Arabica—flour; 3. Arabica—fiber; 4. Robusta Adamantina—extract; 5. Robusta Adamantina—flour; 6. Robusta Adamantina—fiber; 7. Robusta 69.1—flour; 8. Robusta IAC—extract (Instituto Agronômico de Campinas).

### Preparation of extracts

The coffee pulp was extracted by maceration method. Five grams of samples were weighed and subsequently extracted with 100 mL of ethanol 70% (v/v) at 25 °C, under agitation for 20 min and filtered on filter paper^12^. After filtration, solvent was removed from the samples in a rotary evaporator (Fisatom 802) for 1 h, at 80 °C and 150 rpm. After that, the samples were lyophilized in a lyophilizer (Lyostar 3, SP Scientific). The samples were frozen at − 40 °C for 4 h and then dried under vacuum at 100 mTorr. During the drying steps, the extracts went from − 40 to 20 °C, totaling a period of 137 h. The coffee collapse temperature is − 20 °C^13^.

### Quantification of bioactive compounds by HPLC

The lyophilized extracts (10 mg/mL) were solubilized in ethanol 70% (v/v), filtered through a 0.45 μm membrane and content of caffeine and chlorogenic acid was quantified via High Performance Liquid Chromatography (HPLC) using the method previously described by Alves et al.^14^ with modifications. The analysis was performed using HPLC (Lachrom D700, Merck Hitachi, Campinas, Brazil) coupled to a diode array detector (Lachrom L7455, Merck Hitachi, Campinas, Brazil) at 272 nm and 360 nm using a C_18_ chromatographic column (Xterra RP, 5 µm, 3.9 × 150 mm). The mobile phase used was composed by (A) 95% of acetic acid solution (5% v/v, pH 2.4) and (B) 5% of acetonitrile, performed at a constant flow rate of 0.9 mL/min for 15 min, at 25 °C. Data processing was performed at 272 nm for caffeine and 360 nm for chlorogenic acid. Caffeine and chlorogenic acid standards were used to identify the compounds in the samples according to their retention times and to make the analytical curve for quantification. The tests were performed in triplicate^14^.

### Free radical scavenging activity: DPPH

The lyophilized extracts were solubilized in ethanol 70% (v/v) in different concentrations (2.5–10 mg/mL) and 20 µL aliquots were added in a 96-well plate, with 280 µL of 1,1-diphenyl-2-picrilhhydrasyl (DPPH) (32 μg/mL). The samples were incubated in the dark for 30 min at room temperature. After this period, the absorbance was measured at 517 nm in a spectrophotometer (Thermo Scientific, Multiskan Sky). For each concentration of the sample, a blank sample was also prepared, containing 20 µL of the sample and 280 µL of methanol, to discount its absorbance value from the sample, avoiding possible interferences in the reading. A standard curve with gallic acid (0–2 µg/mL) was prepared and the results expressed as percentage of antioxidant activity (% AA) and EC_50_. All tests were performed in triplicate^15^.

### Ferric reducing antioxidant power: FRAP

In the Ferric Reducing Antioxidant Power (FRAP), the lyophilized extracts were solubilized in ethanol 70% (v/v) in different concentrations (2.5–10 mg/mL) and 20 µL aliquots were added in plates of 96 wells with 15 µL of ultrapure water and 265 µL of FRAP reagent. The samples were incubated at 37 °C for 30 min protected from light and the absorbance was measured at 595 nm in a spectrophotometer (Thermo Scientific, Multiskan Sky). For each concentration of the sample, a blank sample was prepared containing 280 µL of ultrapure water and 20 µL of the sample, to discount its absorbance value from the sample, avoiding possible interferences in the reading. A standard curve with gallic acid (0–6 µg/mL) was prepared and the results were expressed as mg gallic acid equivalent/g sample (mg GAE/g sample) and EC_50_. All tests were performed in triplicate^16^.

### Total phenolics

To determine the amount of total phenolic compounds, the Folin-Ciocalteu method was used. The lyophilized extracts were solubilized in ethanol 70% (v/v) (10 mg/mL) and 20 µL aliquots of the samples were added in a 96-well plate with 180 µL of ultrapure water, 20 µL of Folin-Ciocalteu 1 N, 20 µL of methanol and 60 µL of sodium carbonate. Blank sample was also prepared with 20 µL of sample and 280 µL of ultrapure water to be discounted its absorbance from the absorbance of the sample. A standard curve was prepared with gallic acid (0–10 µg/mL). The samples were incubated protected from light for 20 min at room temperature. Absorbances were analyzed on a spectrophotometer (Thermo Scientific, Multiskan Sky) at 760 nm. The results were expressed in mg GAE/g of sample. All tests were performed in triplicate^17^.

### Flavonoids

For the determination of the flavonoid content, 10 mg of the lyophilized extracts were solubilized in 1 mL of ethanol 70% (v/v) and from this solution 100 µL were solubilized in 50 mL of distilled water. 500 µL of this solution was mixed with the same volume as a 2% solution of aluminum chloride (AlCl_3_) in methanol. Blank was prepared using 500 µL of AlCl_3_ with 500 µL of methanol. After 10 min, the absorbance at 425 nm was determined in a spectrophotometer (Thermo Scientific, Multiskan Sky). Flavonoid content was calculated using the standard quercetin curve (20–100 µg/mL). The result was expressed in mg of quercetin equivalents/g sample (mg QE/g sample). All tests were performed in triplicate^18^.

### Tannins

Lyophilized extracts (50 mg) were solubilized in 5 mL of ethanol 70% (v/v) and 1 mL of this solution was mixed with 0.5 mL of the reagent Folin-Ciocalteu, 1 mL of Na_2_CO_3_ solution and 8 mL of distilled water. After 30 min at room temperature, samples absorbance was spectrophotometrically determined at 725 nm (Thermo Scientific, Multiskan Sky). The tannin content was calculated using the tannic acid calibration curve (10–50 mg/mL) result was expressed in mg tannic acid equivalent/L (mg TAE/L). All tests were performed in triplicate^19^.

### Determination of the minimum inhibitory concentration: MIC

In all wells of a 96-well plate, 100 µL of a mixture of 1% (v/v) of DMSO (dimethylsufoxide) and Mueller Hinton agar were deposited. Lyophilized extracts (100 mg) were solubilized in 1 mL of saline and 50 µL of this solution was added to the wells of the first row and serial dilutions were made starting from 50 µL of the first well towards the others. Soon after, 50 µL of the inoculum (0.5 McFarland) was added to each well and the plate was incubated for 24 h at 37 °C. After the incubation period, 40 µL of INT (2-(4-iodophenyl)-3-(4-nitrophenyl)-5-phenyltetrazolium chloride) at 0.2 mg/mL was added to the reaction mixture. The test was performed in triplicate. The microorganisms used were gram positive bacteria *Staphylococcus aureus* (ATCC 10390) and *Streptococcus oralis* (ATCC 9811) and gram negative bacteria *Pseudomonas aeruginosa* (ATCC 9721) and *Escherichia coli* (ATCC 25922)^20^.

### Development of an emulsion containing coffee pulp extract

An oil-in-water (O/W) emulsion was developed according to Table 1, using the standard methods. Two formulations were produced, blank (without extract) and one with 5% coffee pulp extract. All formulation components are natural products approved by the Associação de Certificação Instituto Biodinâmico (IBD), with minimal environmental impact.

**Table 1:** Composition of oil-in water (O/W) emulsion.

Components (INCI NAME)	Function	Composition (%, m/v)
Algin	Hydrophilic thickeners	4.0
Benzoic acid	Preservative	0.3
Glycerin	Humectant	5.0
Lecithin	Surfactant	2.0
Cetyl alcohol	Lipophilic thickeners	4.0
Persea gratissima (avocado) oil	Emollient	5.0
Sorbic acid	Preservative	0.3
Sodium hydroxide solution	pH corrector	q.s pH 5.5–6.5
Aqua	Vehicle	q.s.p 100 mL

### Stability tests

Formulation was evaluated according to stability assay recommended at Stability Guide of Cosmetic Products of ANVISA—Brazilian’s National Health Surveillance Agency^21^. Before the stability tests, 5 g of formulation was submitted to centrifuge cycle at 3000 rpm for 30 min. For the study, formulation was stored in 4 different conditions, room temperature (27 ± 2 °C) protected from indirect light, room temperature (27 ± 2 °C) exposed to indirect light, fridge (5 ± 2 °C) and climatic chamber (45 ± 2 °C).

Preliminary stability of the formulation was carried out for 15 consecutive days, analyzing macroscopic characteristics such as color, odor, appearance and phase separation, besides to pH analysis. Accelerated stability was performed on days 1, 7, 15, 30, 60 and 90 analyzing in addition to the characteristics mentioned above, density using a pycnometer and viscosity in a rotational viscometer (Brookfield, Mod LV-T, São Paulo, Brazil) at 1.5 rpm of rotation for 30 s using spindle 4 at 27 ± 2 °C^21^. The stability test was performed with the formulation blank and with extract, filled in glass containers. To be considered stable, variation in the results should not exceed 10%. The assays were performed in triplicate^21^.

### Cytotoxicity

The cytotoxicity assay was performed using keratinocyte cells (HaCat) and the MTT method (3-bromide (4,5-dimethyl-2-thiazolyl)-2,5 diphenyl-tetrazolium). The cells were cultured in RPMI 1640 medium supplemented with 10% fetal bovine serum and kept in an oven at 37 °C with 5% CO_2_. After cell confluence, the entire bottom of the flask was completed with a cell monolayer, the cells were trypsinized with a solution of 2.5 g/L trypsin/EDTA at 0.2 g/L and transferred to 96-well plates. In this plate, each well received 1 mL of the cell culture at a concentration of 1 × 10^6^ cells/mL and the extracts, blank formulation, phytocosmetic and Market-leading product in wound healing at concentrations of 400, 200, 100, 50, 25, 12.5 and 6.25 µg/mL in triplicate. After 24 h in the oven, the medium was removed and 100 µL of the MTT reagent was added and taken to the oven for 4 h at 37 °C with 5% CO_2_. After this period, the reagent was removed, 100 µL of 100% ethanol was added and the absorbance was read at 570 nm in a spectrophotometer. For the calculation of cell viability, the absorbance value of the treated well was multiplied by 100 and divided by the average absorbance of a control well, which was considered 100%^22^.

### Scratch assay

For the test, HaCat non-tumor cells (keratinocytes) were dispersed across in 12-well plates and incubated for at least 24 h at 37 °C for 5% of CO_2_. Once at convergence, a sterile p200 pipet tip was used to create a straight line of wounds in each well, which was followed by medium removal. Cells were treated with 5% fetal bovine serum (FBS) (positive control), 0.2% fetal bovine serum (negative control), the 8 extracts and the formulation with and without extract. Monitoring of wound closure was observed under an inverted phase microscope at 9, 12, 18, and 24 h. Samples were tested in duplicate, and three images of each well were taken. The images acquired for each sample, at different times, were quantitatively analyzed using ImageJ 1.8.0 software^23^.

### Sensorial analysis

The sensorial analysis was approved by Ethics Committee of University of Campinas (number: 23197519.1.0000.5404). All methods were performed in accordance with relevant guidelines and regulations. All participants agreed to participate in the study, and they provided written informed consent. The study was conducted in accordance with the Declaration of Helsinki. Only blank formulation was subjected to sensorial analysis. Fifty volunteers aged between 18 and 60 old, after signing the informed consent, spreaded 0.1 g of the formulation on the forearm and evaluated the characteristics of speed absorption, speed drying, stickiness, easy spreading, residual fatty sensorial, dry touch, evaluate the product and whether or not to use the formulation, rating each parameter on a scale 1 to 5^24^.

### Statistical analysis

The tests were carried out in triplicate. The results were expressed as mean ± standard deviation and statistical analysis was performed using the ANOVA test followed by Tukey test (p < 0.05) in the GraphPad Prisma 5.0 program. The results for 50% effective concentration (EC_50_) and 50% mean inhibitory concentration (IC_50_) were calculated using Prisma.

## Results and discussion

### Quantification of bioactive compounds by HPLC

Caffeine and chlorogenic acid were identified and quantified in each extract (Table 2). Retention time of caffeine and chlorogenic acid were similar in extracts and in the standard, with caffeine appearing in 4.26 min and chlorogenic acid 7.67 min. Samples 1, 2 and 3 of arabica species had a higher caffeine content than those of robusta species, an opposite result compared with Sunarharum et al.^25^. The highest chlorogenic acid content was found in samples of robusta species, confirming a previous study^26^. Therefore, the best extract for caffeine was sample 2, and the best for chlorogenic acid was extract 8, as it showed the highest concentration of these compounds.

**Table 2:** Concentration of caffeine and chlorogenic acid (µg/mL) in coffee samples.

Samples	Caffeine	Chlorogenic acid
1	190.02	55.56
2	338.17	14.50
3	317.88	21.88
4	27.83	52.43
5	114.59	82.52
6	126.02	156.74
7	76.13	17.97
8	79.40	192.92

Literature indicates that in green coffee beans the content of chlorogenic acid can vary from 61.15 ± 1.40 to 86.42 ± 2.04 mg/g, based on wet matter^27^ and caffeine from 10.5 to 19.9 mg/g^28^. The pulp showed a smaller number of active compounds than in the coffee bean, which is normal once it is a residue, however even with the amount found in this work, the samples showed satisfactory antioxidant and antibacterial activities, as will be discussed below.

### Free radical scavenging activity: DPPH

Analyzing the antioxidant properties of extracts is essential because of its direct association with the healing process of wounds. Injuries can trigger oxidative stress, resulting in the production of reactive oxygen species (ROS) that may damage cellular structures. Antioxidants play a crucial role in counteracting these ROS, providing effective protection for cells against oxidative damage^29^.

The results obtained are shown in Table 3. All extracts showed antioxidant activity values greater than 81% at 10 mg/mL concentration. The best results were observed for samples 2 (95.45 ± 0.10%), 6 (92.51 ± 0.58%) and 5 (92.33 ± 0.48%) with no significant difference between them. These results may be related to active compounds amount present in samples, once extracts 2 and 6 had the highest concentration of caffeine and chlorogenic acid, respectively. EC_50_ is the required effective concentration of antioxidants to reduce the initial concentration of free radical by 50%. Therefore, the lower the EC_50_, the better the performance of the sample. Thus, the extract that obtained the best EC_50_ result was sample 5 (2.57 mg/mL).

**Table 3:** Antioxidant activity evaluation of coffee samples (10 mg/mL) using DPPH (2,2-diphenyl-1-picrylhydrazyl) and FRAP (ferric reducing antioxidant power) assays.

Samples	DPPH		FRAP		Total phenolics	Flavonoids	Tannins
	%AA	EC_50_ mg/mL	mg GAE/g sample	EC_50_ mg/mL	mg GAE/g sample	mg QE/g sample	mg TAE/L
1	83.64 ± 2.43^ad^	4.07	7.28 ± 0.20^ab^	5.12	20.05 ± 1.68^a^	47.33 ± 3.85^ad^	47.95 ± 2.21^a^
2	95.45 ± 0.10^b^	4.10	8.93 ± 0.62^a^	7.12	19.46 ± 0.82^a^	46.22 ± 13.85^a^	44.47 ± 0.33^a^
3	87.00 ± 3.78^ae^	5.08	5.48 ± 0.25^b^	5.80	10.75 ± 0.43^b^	21.78 ± 3.33^b^	31.97 ± 0.29^b^
4	88.62 ± 0.21^ace^	4.99	34.11 ± 0.31^c^	6.33	33.04 ± 2.76^c^	32.89 ± 1.93^c^	89.75 ± 2.27^c^
5	92.33 ± 0.48^bce^	2.57	8.42 ± 0.21^ab^	7.58	17.31 ± 1.53^ab^	25.11 ± 3.33^bc^	45.07 ± 0.75^a^
6	92.51 ± 0.58^bce^	–	40.66 ± 0.73^d^	6.48	44.22 ± 3.58^d^	42.89 ± 1.93^a^	110.94 ± 3.10^d^
7	81.55 ± 1.91^d^	6,27	4.89 ± 0.14^b^	7.26	8.31 ± 0.50^b^	55.11 ± 3.33^d^	22.73 ± 2.22^e^
8	89.81 ± 1.41^e^	–	58.98 ± 2.88^e^	4.51	79.53 ± 5.61^e^	46.22 ± 1.92^a^	133.81 ± 3.56^f^

The results are derived as mean ± standard deviation (n = 3) and the EC_50_ value.

*AA* antioxidant activity, *GAE* gallic acid equivalent, *EC*_*50*_ half maximal effective concentration, *GAE* gallic acid equivalent, *QE* quercetin equivalent, *TAE* tannic acid equivalent.

Different letters in the same column indicate significant differences according to one-way ANOVA (p < 0.05) and Tukey’s test.

Results obtained in this study were like those found by Andrade et al.^30^ who obtained 91.5% and 90.3% of antioxidant activity for coffee husk extracted by ultrasound and Soxhlet, respectively. In addition, it was greater than or equal to that observed by Palomino García et al.^31^ who obtained values for peel from 34.8 to 82.6% antioxidant activity and for pulp 26.0 to 77.9% antioxidant activity. Therefore, the results indicate a promising antioxidant activity in the studied samples.

### Ferric reducing antioxidant power: FRAP

The results are shown in Table 3. Sample 8 at a concentration of 10 mg/mL showed the best results with 58.98 ± 2.88 mg GAE/g sample and EC_50_ of 4.51 mg/mL. Interestingly, sample 8 was also the sample with the highest concentration of chlorogenic acid, to whom is attributed antioxidant properties^32^.

In the existing literature, there is no documented research specifically examining the Ferric Reducing Antioxidant Power (FRAP) of coffee using the unit of mg GAE/g sample. However, a distinct study centered on coffee pulp assessed the FRAP using the unit of mg FeSO_4_/g sample, resulting in a value of 10.90 ± 0.24 mg FeSO_4_/g sample^33^. Due to the discrepancy in measurement units, making a direct comparison between these findings poses a significant challenge.

### Total phenolics

Phenolic compounds are secondary metabolites present in several plants, that produce beneficial activities, such as antioxidant activity depending on their ability to donate electrons^34^. Values found ranged from 8.31 ± 0.50 to 79.53 ± 5.61 mg GAE/g sample for samples in 10 mg/mL concentrations, with extract 8 showing the best result (Table 3). These values are in accordance with results obtained by previous studies that showed 104.3 mg GAE/g for raw coffee^35^ and 17.75 mg GAE/g for spent coffee grounds 1 and 21.56 mg GAE/g for spent coffee grounds 2^36^. Therefore, we can conclude that in the residues a significant amount of compounds of interest can also be found.

### Flavonoids

Flavonoids are the group of phenols most found in plants with high antioxidant efficacy. They inhibit metal-initiated lipid oxidation and form complexes with metal ions^37^. Flavonoid’s concentrations found are shown in Table 3 and ranged from 21.78 ± 3.33 to 55.11 ± 3.33 mg QE/g sample. The highest flavonoid content was observed in sample 7 (55.11 ± 3.33 mg QE/g sample), but it did not differ statistically from sample 1 (47.33 ± 3.85 mg QE/g sample) and the sample with the lowest content was sample 3 (21.78 ± 3.33 mg QE/g sample), which did not differ statistically from sample 5 (25.11 ± 3.33 mg QE/g sample). Arabica and robusta presented the best and worst results, thus flavonoid content may not be related to coffee species, but rather to other factors, such as extraction mode, collection period, or collection region.

Results found in this study using coffee pulp were superior to those published by Kreicbergs et al.^38^ using coffee bean, with values of approximately 18 to 105 mg QE/100 g. This fact highlights once again the importance of taking advantage of these by-products that are discarded.

### Tannins

Tannins are complex compounds that contain polyphenols with high biological activity and properties as anti-inflammatory^39^ and antibacterial^40^. Therefore, a product containing coffee can be very beneficial in the healing process. Tannins concentration ranged from 22.73 ± 2.22 to 133.81 ± 3.56 mg TAE/L (Table 3). The highest content of tannins obtained was for sample 8 and the lowest for sample 7. Most of the samples of robusta species obtained better results than the samples of arabica species.

Previous studies have found a maximum tannin content of 0.43 ± 0.06 mg TAE/L for silverskin and 0.61 ± 0.04 mg TAE/mL in spent coffee ground^41^, so it can be inferred that the pulp has a greater amount of tannins than the silverskin and spent coffee ground.

### Determination of the minimum inhibitory concentration: MIC

The minimum inhibitory concentration is the lowest concentration of an antimicrobial capable of inhibiting a microorganism visible growth after 24 h of incubation^42^. For this test, samples 2 and 8 were analyzed (Table 4), once they had the highest concentration of caffeine, chlorogenic acid, and the best antioxidant activity. Sample 8 showed the best antibacterial activity against *Staphylococcus aureus* with MIC of 3 mg/mL. Sample 2 showed better inhibition of *Pseudomonas aeruginosa* with MIC of 12.5 mg/mL. Hence, sample 8 is deemed the most effective, as it demonstrated the lowest concentration value in inhibiting the bacteria.

**Table 4:** Minimum inhibitory concentration (MIC) in mg/mL of coffee extracts at 100 mg/mL.

Microrganismos		Sample 2	Sample 8
Gram positive	*Staphylococcus aureus*	25	3
	*Streptococcus oralis*	25	25
Gram negative	*Pseudomonas aeruginosa*	12.5	12.5
	*Escherichia coli*	25	–

The research conducted by Rawangkan et al.^43^ delved into the Minimum Inhibitory Concentration (MIC) assessment of dried green coffee beans, coffee pulp, and arabica leaf crude extracts against 28 strains of multidrug-resistant *E. coli*. The study also investigated the restoration of ampicillin efficacy through a combination test. Results indicated that dried green coffee, coffee pulp, and arabica leaf extracts were effective against all 28 strains, with a MIC range of 12.5–50 mg/mL. Notably, combining coffee pulp with ampicillin demonstrated superior efficacy compared to using either substance alone, with a fractional inhibitory concentration index value of 0.01. In this combination, the MIC of coffee pulp decreased to 0.2 mg/mL (as opposed to 25 mg/mL when using coffee pulp alone), and the MIC of ampicillin decreased to 0.1 mg/mL (in contrast to 50 mg/mL for ampicillin alone). Consequently, Sample 2 in our study produced comparable results, with a MIC of 25 mg/mL against *E. coli*. This aligns with the aforementioned study, providing additional evidence for the significant antimicrobial activity of coffee by-products.

### Development and stability of an emulsion containing coffee pulp extract

The chosen pharmaceutical form was an emulsion because it provides a stable and homogeneous system for integrating active ingredients. Considering the complex composition of coffee pulp extract, an emulsion serves as a dependable platform to maintain the stability of these bioactive components over time. Furthermore, emulsions create an appropriate environment for solubilizing both hydrophilic and lipophilic compounds. The utilization of an emulsion allows for the generation of smaller particle sizes, facilitating improved penetration of bioactive compounds into the skin. This is essential for achieving efficient absorption and promoting the desired antioxidant and antimicrobial effects. Additionally, emulsions offer ease of application, can be tailored for controlled release of active ingredients, and can be formulated to present an appealing appearance and texture, thereby enhancing the overall user’s sensory experience.

Sample 8 was selected to be incorporated into the emulsion, as it presented the best results among the other extracts, in terms of chlorogenic acid concentration, FRAP, total phenolics and antibacterial assay Two formulations were developed, one blank (without extract) and the phytocosmetic (with extract). The formulation was white, creamy, shiny, with a characteristic odor of base formulation and in the presence of the extract, had a slightly yellow color. Blank emulsions and phytocosmetic had skin compatible pH (6.17 and 6.02), density of 0.96 g/mL which indicates a low amount of air incorporated in the formulation during its preparation and viscosity of 47.333 ± 1.155 cP and 51.333 ± 2.309 cP, respectively. As mentioned in the literature, characteristics found in the formulations are in accordance with the requirements for topical products^44^.

The formulations stored at 45 ± 2 °C in a climatic chamber, darkened and phase separation occurred on the seventh day, which could be attributed to decomposition reactions, as oxidative process, accelerated by elevated temperatures accelerate decomposition reactions like oxidative processes^45^.

In the other conditions, phase separation was not observed in centrifugation test and formulations kept the color, odor, and appearance stable for 90 days. Formulations containing extract stored at room temperature protected and exposed to light remained stable during the 90 days. The viscosity had a variation greater than 10% for blank formulation stored at room temperature exposed to light and at fridge on the 60th day and the blank formulation stored in the climatic chamber and the one with extract in the fridge on the seventh day. Finally, all formulations showed stable density with less than 10% variation. Throughout the period of accelerated stability, these results showed that the formulation with extract stored at room temperature, both protected and exposed to light, remained stable.

### Cytotoxicity

**Figure 1:**
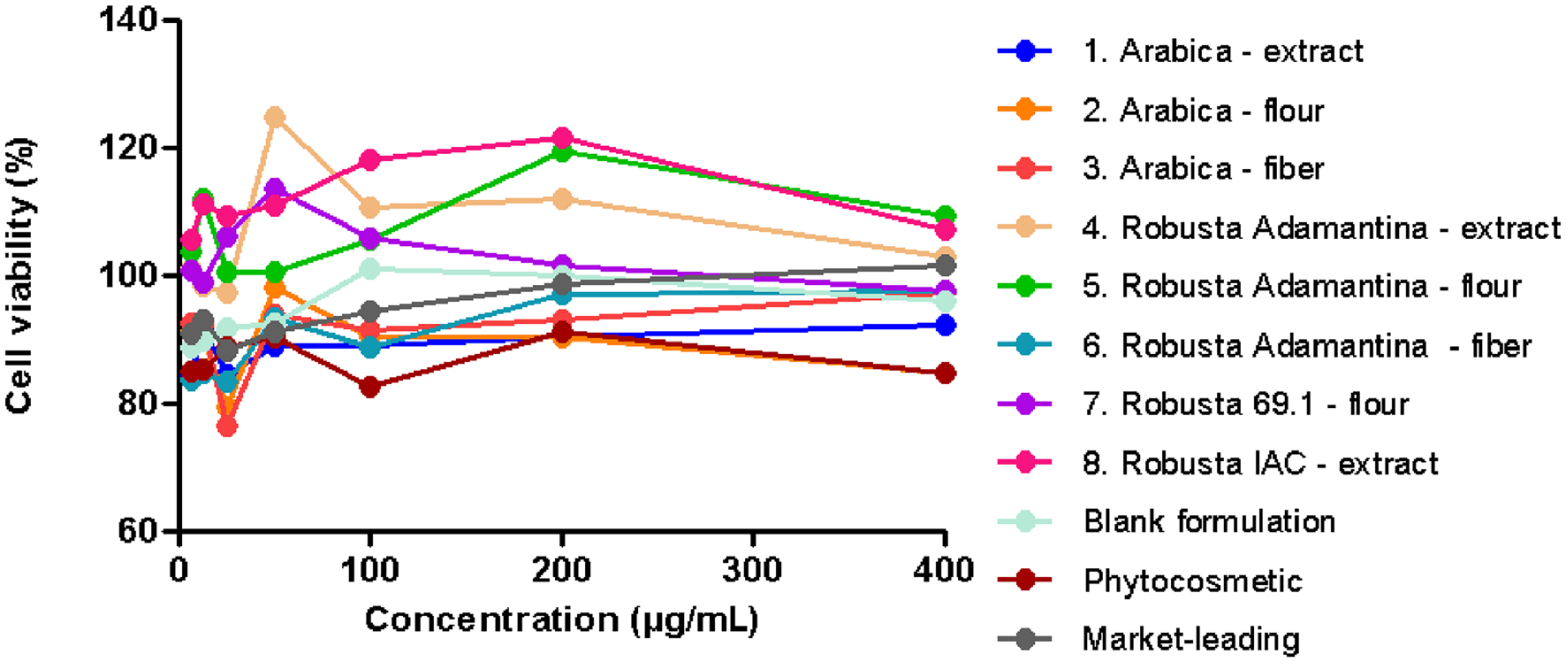
HaCat (human non-tumor keratinocyte) viability after 24 h of incubation with coffee extracts, obtained by the MTT method at 570 nm.

Samples tested in different concentrations showed cell viability ≥ 76%, indicating that the extracts and formulations do not have cytotoxicity against the used cells, as a sample is considered cytotoxic when it reduces more than 70% in cells viability^46^. Sample 4 showed the highest viability reaching 124.85 ± 23.28% at a concentration of 50 µg/mL (Fig. 1). Samples of arabica species caused lower cell viability compared to robust. Another study in the literature reported that an ethanolic extract of coffee silverskin was cytotoxic at concentrations up to 400 µg/mL using HaCat cells^47^. All samples IC_50_ ranged from 0.03 to 159.1 µg/mL: 1–46.80 µg/mL; 2–159.1 µg/mL; 3–7.748 µg/mL; 4–288.5 µg/mL; 5–51.72 µg/mL; 6–73.69 µg/mL; 7–0.03 µg/mL; 8–122.3 µg/mL, with best results for samples 4, 2 and 8, since the IC_50_ is the concentration of the substance required to inhibit the function by 50%. Several works also report that coffee residues are usually safe because they did not show cytotoxicity, even at high concentrations^48–50^. Therefore, the extracts and formulations were not toxic to keratinocytes at the concentrations tested, being a first indication that it would be safe for topical use.

### Scratch assay

**Figure 2:**
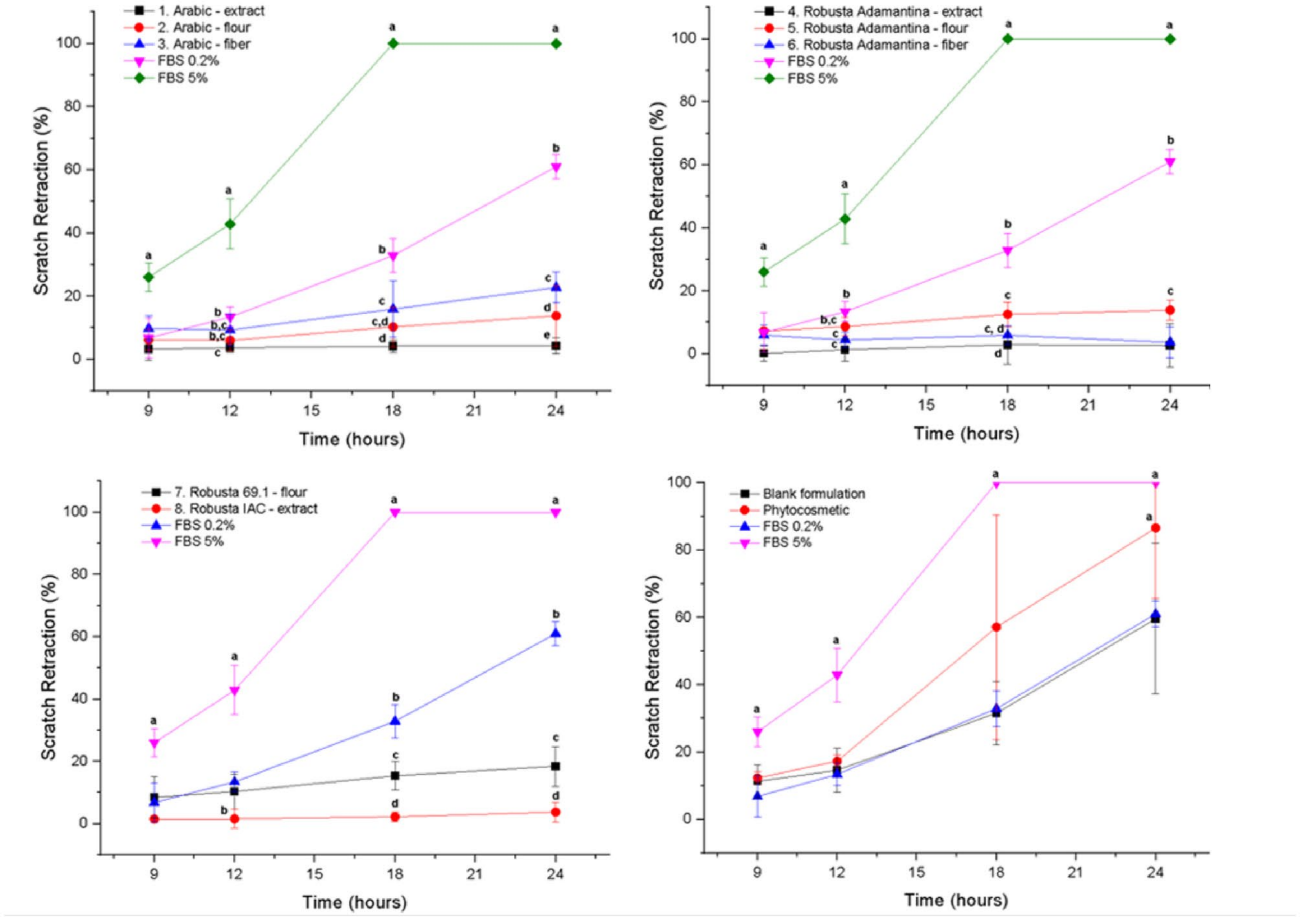
Scratch retraction percentage of controls and samples on scratch retraction during assay period. FBS:fetal bovine serum. Data is presented as mean ± standard deviation; n = 4. Letters represent statistical significance when comparing treatments in the same time point by Tukey’s test.

Extracts inhibited cell migration in the conditions used (cells kept in 0.2% of fetal bovine serum). Blank formulation did not affect cellular migration, and showed no significant difference with negative control (Fig. 2). However, phytocosmetic promoted cell migration after 24 h and showed no significant difference with positive control. Therefore, the formulation with the extract increased scratch retraction, thus indicating that it could be used in a healing process.

One study investigated the impact of 2.5% and 5% *Coffea canephora* bean extract on the migration of fibroblasts for wound healing. The extracts induced a significant increase in the migration of NIH3T3 cells/fibroblasts after 24 h and 48 h, as evidenced by a reduction in the widths of the scratched areas^51^. Thus, another study shows that coffee has the potential to be used in wound healing.

### Sensorial analysis

**Figure 3:**
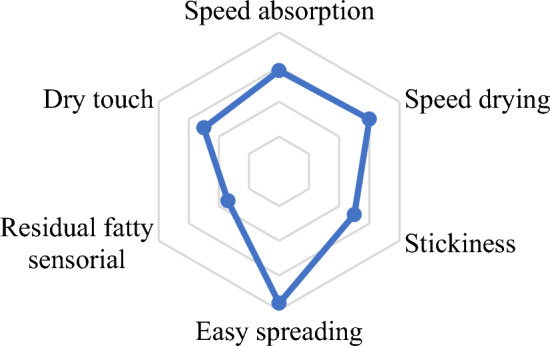
Evaluation of aspects such as speed absorption, speed drying, stickiness, spreading, residual fatty sensorial and dry touch of blank formulation by sensorial analysis.

Among the 50 volunteers (19 men and 31 women) who participated in the sensory analysis, 44 (88%) said they would use the product and the average rating of the overall formulation was 4.26 on a scale from 1 to 5. The formulation faded, a desirable characteristic for topical products, the formulation also dried fast, it was not sticky, spreaded easily, highest score among parameters; the residual fatty sensorial was low. Overall, formulation had a pleasant good dry touch. Therefore, the formulation was well accepted by the study participants, as all parameters obtained scores close to the maximum score of 5 (Fig. 3), showing that the developed formulation has great chances of being accepted by consumers in the final market.

Another study that developed phytocosmetic using only organic components also showed good results in terms of absorption speed, drying, easy spreading, low residual fatty sensation, and stickiness, with a dry touch^24^. This confirms that formulations with natural ingredients are well-received by consumers.

## Conclusion

Coffee pulp extract, an abundant by-product in the agribusiness and most often discarded or used as fertilizer, showed satisfactory results in the performed tests. Among the samples analyzed, Robusta IAC—extract (sample 8), stood out with the best results in FRAP antioxidant assay, total phenolics, higher chlorogenic acid content, antibacterial activity, and less cytotoxic potential. Thus, this extract was incorporated into a phytocosmetic that was stable at room temperature for a period of 90 days, promoted cell migration, consequently improved the healing process. The blank formulation was well accepted by volunteers in the sensory analysis. Therefore, it was possible to achieve the goal of this study by developing a phytocosmetic using only natural products containing coffee pulp extract as the active pharmaceutical ingredient, that presented characteristics that indicate a promising application due to its antioxidant, antimicrobial and healing properties.

## Data Availability

All data produced and/or examined throughout this research are incorporated within this published paper.
